# Bibliographical—Tin Foil and Its Combinations for Filling Teeth

**Published:** 1897-12

**Authors:** 


### EDITORIAL.

### Bibliographical.

TIN FOIL, AND ITS COMBINATIONS FOR FILLING TEETH. By Henry L. Ambler, M. S., D. D. S., M. D., Professor of Operative Dentistry and Dental Hygiene in the Dental Department of Western Reserve University, etc., etc.

The subject of this little treatise is one that has not received the attention and consideration that is justly its due. It, in many respects, is the most valuable material for filling teeth, now in use, and one, the true value of which is largely overlooked, or rather, neglected. There are many cases in which nothing is equal to it as a filling material; as in the teeth of children, and those of the softer varieties; and in many cases, it, with gold, accomplishes results that can not be attained with gold alone. In almost every instance in which amalgam is used, tin would serve a better purpose; indeed, it ought to supplant amalgam wholly; and in a great many cases in which other plastics are used, tin would be much better, and especially where comparatively permanent results are sought. Tin as a foundation for gold filling is valuable, and mixed with gold is highly prized by many. This mixture was first brought to the attention of the profession by the late Dr. Abbott, of Berlin, Germany.

Dr. Ambler has rendered the dental profession an invaluable service in the preparation of this work. He probably has no superior, if an equal, in working tin as a material for filling teeth. It is a subject to which he has devoted special attention throughout his professional life, a period of about thirty years. Not only the experience of Dr. Abbott is here given, but he has presented the experience of many other prominent operators in this country.

It is to be hoped that this work will be an incentive to many who use tin but little or not at all, to use it more and more, and secure all the benefit that may be derived from its skillful use. The book ought to be in the hands of every dentist in the country, and especially should it be in the possession of every student as soon as he begins to operate on the natural teeth.

The work is published by the S. S. White Dental Manufacturing Co. It is neat in form and well gotten up in every respect.

